# Environmental DNA assays for the sister taxa sauger (*Sander canadensis*) and walleye (*Sander vitreus*)

**DOI:** 10.1371/journal.pone.0176459

**Published:** 2017-04-25

**Authors:** Joseph C. Dysthe, Kellie J. Carim, Michael Ruggles, Kevin S. McKelvey, Michael K. Young, Michael K. Schwartz

**Affiliations:** 1 United States Department of Agriculture, Forest Service, National Genomics Center for Wildlife and Fish Conservation, Rocky Mountain Research Station, Missoula, Montana, United States of America; 2 Montana Department of Fish, Wildlife and Parks, Region 5, Billings, Montana, United States of America; National Cheng Kung University, TAIWAN

## Abstract

Sauger (*Sander canadensis*) and walleye (*S*. *vitreus*) are percid fishes that naturally co-occur throughout much of the eastern United States. The native range of sauger extends into the upper Missouri River drainage where walleye did not historically occur, but have been stocked as a sport fish. Sauger populations have been declining due to habitat loss, fragmentation, and competition with non-native species, such as walleye. To effectively manage sauger populations, it is necessary to identify areas where sauger occur, and particularly where they co-occur with walleye. We developed quantitative PCR assays that can detect sauger and walleye DNA in filtered water samples. Each assay efficiently detected low quantities of target DNA and failed to detect DNA of non-target species with which they commonly co-occur.

## Introduction

Sauger (*Sander canadensis*) and walleye (*S*. *vitreus*) are sister taxa in the family Percidae that naturally co-occur in cool-water habitats throughout much of central and eastern North America [1–2]. Historically, walleye were more widely distributed throughout this region, whereas sauger were more limited, but found farther west [1]. Across their overlapping geographic range, walleye occur in a greater variety of lentic and riverine habitats and tolerate a wider array of water quality conditions, while sauger are typically limited to large, turbid systems [3]. In areas where both species naturally coexist, sympatry is maintained through temporal and spatial separation within the system as sauger spawn later and prefer greater depths than walleye [3–4]. However, interspecific competition may be higher where habitat has been altered or in areas where sauger and walleye did not historically co-occur [5]. Because both species are prized as gamefish, each has been stocked well outside its historical range, often in locations inhabited by the other species.

In the U.S., some populations of sauger have declined due to habitat loss, alteration, fragmentation, and exploitation [6–9]. The development of dams and reservoirs can destroy spawning habitat, alter flow regimes, and impact water clarity, which has been attributed to declines of sauger in the upper Missouri River [8]. Furthermore, this trend may have been exacerbated by competition or hybridization following introductions of walleye where the two species historically did not co-occur [5, 10–11]. For example, upstream of the Fort Peck Dam on the Missouri River, sauger have experienced a significant decline in abundance relative to an established population of introduced walleye [6]. Although this localized decline of sauger is attributed to drought in the 1980’s [6], the species’ inability to recover may be in large part due to the competitive pressures exerted by the non-native walleye [5]. Consequently, understanding the distribution of both species is a conservation priority for fisheries managers. Finding existing populations that remain in low abundance may provide a method to prioritize recovery actions. Since both species occupy habitats that are often challenging to sample effectively using traditional sampling methods, detecting accurate changes in their distributions is often problematic.

In many habitats, environmental DNA (eDNA) is emerging as a reliable and highly sensitive alternative sampling method for detecting the occurrence and distributions of aquatic species [12–16], even among closely related taxa [17]. When coupled with quantitative PCR (qPCR) technology, eDNA analysis has proven to be more sensitive than traditional PCR methods in detecting low concentrations of targeted DNA [18]. Here, we describe separate eDNA assays specific to sauger and walleye that can be employed quickly and reliably to help managers understand the distribution of these species.

## Methods

We designed TaqMan^™^ assays with minor-groove-binding probes (TaqMan MGB; Applied Biosystems—Life Technologies Corporation) targeting mitochondrial markers specific to sauger or walleye. For sauger, we compiled GenBank DNA sequences of the whole mitochondrial genome and cytochrome *b* (cyt*b*) gene, along with published sequences for closely related or potentially sympatric species (Table 1). The sauger sequences were from fish originating from seven locations; Mississippi River in IL [19], Tennessee River in TN [20], Arkansas River in AR, Perry Lake in KS, Lake of the Woods in MN, Lake Wisconsin in WI [2], and across eastern Ontario in Canada [21]. For walleye, we compiled GenBank sequences of the whole mitochondrial genome and NADH dehydrogenase subunit 2 (ND2) gene along with published sequences for closely related species and those overlapping in distribution (Table 1). Location information was not available for walleye sequences. Using the *DECIPHER* package [22] in R v. 3.0.3 [23], we screened the sequences *in silico* and obtained candidate primers unique to each target species. We aligned the primers with the sequence data in MEGA 6.0 [24], manually adjusted them to maximize base pair mismatches with non-target species, and optimized annealing temperatures by modifying primer lengths in Primer Express 3.0.1 (Life Technologies; Table 2). The primers amplify a 112- and 175-base-pair fragment in sauger and walleye respectively. There are at least seven base-pair mismatches between the sauger primer pair and non-target DNA sequences (Table 1), and at least 16 base-pair mismatches between the walleye primer pair and non-target DNA sequences (Table 1).

**Table 1:** Species, samples size (*n*), and GenBank accession number for DNA sequences used for *in silico* development of eDNA markers for sauger and walleye. Also included is the minimum number of base pair mismatches between each component of markers and the sequences screened.

Marker	Common name	Family	Species	*n*	GenBank accession	Forward primer mismatches	Reverse primer mismatches	Probe mismatches
Sauger (cyt*b*)	Sauger	Percidae	*Sander canadensis*	19	AF386603.1; AY374290.1; DQ451391.1 –DQ451400.1; KC819814.1 –KC819818.1; KT211477.1 –KT211478.1	0	0	0
	Channel catfish	Ictaluridae	*Ictalurus punctatus*	2	AY458886.1; EU490914.1	8	6	4
	Common carp	Cyprinidae	*Cyprinus carpio*	2	DQ868875.1; KF574487.1	5	7	7
	Common logperch	Percidae	*Percina caprodes*	2	EU379094.1-EU379095.1	6	5	3
	Crystal darter	Percidae	*Crystallaria asprella*	1	AF099903.1	6	4	5
	Flathead chub	Cyprinidae	*Platygobio gracilis*	2	EU811100.1; JX442992.1	6	4	4
	Freshwater drum	Sciaenidae	*Aplodinotus grunniens*	2	AY225662.1; KP722606.1	8	7	6
	Goldeye	Hiodontidae	*Hiodon alosoides*	1	AY504821.1	7	10	7
	Meramec saddled darter	Percidae	*Etheostoma erythrozonum*	1	HQ128170.1	5	6	7
	Northern pike	Esocidae	*Esox lucius*	2	AY497445.1; AY497452.1	6	6	6
	River carpsucker	Catostomidae	*Carpiodes carpio*	2	JF799431.1; JN053258.1	4	5	6
	Shorthead redhorse	Catostomidae	*Moxostoma macrolepidotum*	2	JF799473.1; JF799476.1	6	7	6
	Shovelnose sturgeon	Acipenseridae	*Scaphirhynchus platorynchus*	2	SPU56984.1; SPU56988.1	8	7	4
	Smallmouth bass	Centrarchidae	*Micropterus dolomieu*	2	HM070845.1; HM070903.1	6	8	5
	Stonecat	Ictaluridae	*Noturus flavus*	2	AY458892.1; KM264121.1	7	8	4
	Walleye	Percidae	*Sander vitreus*	6	AF045359.1; AF386602.1; KC819819.1 –KC819822.1	5	4	2
	Western sand darter	Percidae	*Ammocrypta clara*	1	HQ128065.1	5	2	4
	Western silvery minnow	Cyprinidae	*Hybognathus argyritis*	2	EU811093.1 –EU811094.1	5	7	6
	White sucker	Catostomidae	*Catostomus commersonii*	2	JF799435.1; JF799437.1	6	5	6
	Yellow perch	Percidae	*Perca flavescens*	2	AF043557.1; KC819830.1	8	7	2
Walleye (ND2)	Walleye	Percidae	*Sander vitreus*	62	FJ381257.1; JQ088644.1 –JQ088645.1; KP013098.1; KT211421.1 –KT211476.1	0	0	0
	Channel catfish	Ictaluridae	*Ictalurus punctatus*	1	AF482987.1	9	10	7
	Common carp	Cyprinidae	*Cyprinus carpio*	1	KF856965.1	10	9	5
	Common logperch	Percidae	*Percina caprodes*	2	EU379080.1-EU379081.1	13	10	4
	Crystal darter	Percidae	*Crystallaria asprella*	1	JQ088502.1	13	10	4
	Freshwater drum	Sciaenidae	*Aplodinotus grunniens*	1	AY225720.1	9	10	4
	Goldeye	Hiodontidae	*Hiodon alosoides*	1	AP004356.1	8	8	4
	Meramec saddled darter	Percidae	*Etheostoma erythrozonum*	1	JQ088594.1	11	11	5
	River carpsucker	Catostomidae	*Carpiodes carpio*	1	AP006763.1	9	12	4
	Sauger	Percidae	*Sander canadensis*	2	JX088642.1; KC663435.1	10	8	2
	Shorthead redhorse	Catostomidae	*Moxostoma macrolepidotum*	1	JX488921.1	12	9	5
	Smallmouth bass	Centrarchidae	*Micropterus dolomieu*	1	AY225753.1	11	10	6
	Western sand darter	Percidae	*Ammocrypta clara*	1	EF027172.1	13	11	4
	White sucker	Catostomidae	*Catostomus commersonii*	1	JX488850.1	11	10	5
	Yellow perch	Percidae	*Perca flavescens*	1	AY225721.1	13	10	4

**Table 2:** Primers and probes to detect sauger and walleye using qPCR.

Assay Component	Sequence (5’-3’)	Tm (°C)	Final concentration (nM)
Sauger forward primer	TGGGGTCATCCTCCTTCTRAT	56.2–59.3	900
Sauger reverse primer	TGCAGATAAGAGGTTAGTAATGACGGTA	59.5	900
Sauger probe	FAM-TTTGTAGGGTATGTATTACCCTGA-MGBNFQ	69.0	250
Walleye forward primer	CTATTATACTATTTACCCTCGGGCTCG	59.7	600
Walleye reverse primer	GTCGATTGAACAATGAAGTATTTTGC	59.0	600
Walleye probe	FAM-TAATTGCCTGAATGGGTC-MGBNFQ	69.0	250

Using the MEGA sequence alignments, we visually identified species-specific regions between the primers and designed a TaqMan MGB probe (Applied Biosystems) with 6-carboxyfluorescein (FAM)-labeled 5’ ends and minor-groove-binding, non-fluorescent quenchers (MGB-NFQ) for each species (Table 2). There are a minimum of two base-pair mismatches with each probe and any non-target species. We assessed annealing temperature of each probe in Primer Express 3.0.1 (Life Technologies; Table 2) and screened each primer-probe set for secondary structures using IDT OligoAnalyzer (https://www.idtdna.com/calc/analyzer). To confirm the specificity of each assay *in silico*, we performed BLAST searches on each primer and probe sequence.

To test the specificity of each assay *in vitro*, we screened DNA extracted from tissue of each target species and from non-target species with which they commonly co-occur. For the sauger assay, we screened DNA of 23 sauger from 21 locations in five drainages throughout Montana and 31 additional non-target species (Table 3). For the walleye assay, we screened DNA of 19 walleye from 15 locations in 12 drainages throughout the U.S., and 31 additional non-target species (Table 3). The samples used in this study were from archived DNA and tissues collected during previous studies. As such, approval by an animal ethics committee was not required. All sauger and most walleye tissues were obtained from archived samples at the Montana Fish, Wildlife and Parks Conservation Genetics Laboratory collected for previous studies (see [25]). Tissue from Minnesota walleye were provided by the Minnesota Department of Natural Resources from fish collected during surveys conducted in 2015. Likewise, non-target tissues were provided by Montana Fish, Wildlife, and Parks from fish collected during surveys conducted in 2015. Tissues were obtained by excising a small fin clip and releasing the fish at the point of capture. All tissues were stored in 95% ethanol until DNA extraction and DNA was extracted using the DNeasy Tissue and Blood Kit (Qiagen, Inc) using the manufacturer’s protocol.

**Table 3:** Species used for *in vitro* testing of the primers and probe. Source refers to the waterbody for sauger and walleye specimens. For all other specimens, source is listed by state.

		Specimens tested (*n*)		
Common name	Species	Sauger	Walleye	Source
Sauger	*Sander canadensis*	5	4	Bighorn River basin, WY
		3	1	Milk River, MT
		4	4	Missouri River basin, MT
		1	1	Tongue River, MT
		10	6	Yellowstone River basin, MT
Walleye	*Sander vitreus*	1	1	Lake Erie, OH
		1	1	Cumberland Drainage, KY
		1	1	Tongue River, MT
		1	1	Yellowstone River, MT
		1	3	Mississippi River, MN
		1	2	Hudson River, MN
		1	3	Lake Gogebic, MI
		1	1	Muskegon River, MI
		1	1	Lake Mistassini, QC
		1	3	Lake Washington, WA
		1	1	Columbia River, WA
		1	1	Bighorn Lake, WY
Black crappie	*Pomoxis nigromaculatus*	1	1	MT
Brown trout	*Salmo trutta*	1	1	MT
Burbot	*Lota lota*	1	1	MT
Channel catfish	*Ictalurus punctatus*	1	1	MT
Common carp	*Cyprinus carpio*	1	1	MT
Emerald shiner	*Notropis atherinoides*	1	1	MT
Fathead minnow	*Pimephales promelas*	1	1	MT
Flathead chub	*Platygobio gracilis*	1	1	MT
Freshwater drum	*Aplodinotus grunniens*	1	1	MT
Goldeye	*Hiodon alosoides*	1	1	MT
Largemouth bass	*Micropterus salmoides*	1	1	MT
Longnose dace	*Rhinichthys cataractae*	1	1	MT
Longnose sucker	*Catostomus catostomus*	1	1	MT
Mountain sucker	*Catostomus platyrhynchus*	1	1	MT
Northern pike	*Esox lucius*	1	1	MT
Rainbow trout (inland steelhead)	*Oncorhynchus mykiss gairdneri*	1	1	ID
River carpsucker	*Carpiodes carpio*	1	1	MT
Sand shiner	*Notropis stramineus*	1	1	MT
Shorthead redhorse	*Moxostoma macrolepidotum*	1	1	MT
Shovelnose sturgeon	*Scaphirhynchus platorynchus*	1	1	MT
Sicklefin chub	*Macrhybopsis meeki*	1	1	MT
Smallmouth bass	*Micropterus dolomieu*	1	1	MT
Smallmouth buffalo	*Ictiobus bubalus*	1	1	MT
Stonecat	*Noturus flavus*	1	1	MT
Sturgeon chub	*Macrhybopsis gelida*	1	1	MT
Western silvery minnow	*Hybognathus argyritis*	1	1	MT
White crappie	*Pomoxis annularis*	1	1	MT
White sucker	*Catostomus commersonii*	1	1	MT
Yellow bullhead	*Ictalurus natalis*	1	1	MT
Yellow perch	*Perca flavescens*	2	2	MT, WA

We tested each qPCR assay with a StepOne Plus Real-time PCR Instrument (Life Technologies) in 15-μl reactions containing 7.5 μl Environmental Master Mix 2.0 (Life Technologies), 900 nM of each primer, 250 nM probe, 4 μl DNA template (~0.1–1.0 ng), and 2.75 μl deionized water. Thermocycler conditions are as follows: initial denaturation for 10 min at 95°C followed by 45 cycles of denaturation for 15 s at 95°C and annealing for 1 min at 60°C. Each test included a no-template control with distilled water used in place of DNA template; all qPCR tests were set up inside a hood where pipettes, tips, and set-up tubes were irradiated with UV light for 1 h before each test.

We optimized primer concentrations (Table 2) in each assay by varying concentrations of each primer (100, 300, 600, and 900 nM) for a total of 16 different combinations [17]. We then tested the sensitivity of each assay using the optimized assay concentrations and cycling conditions by performing standard curve experiments created from target qPCR product. The qPCR product was purified using PureLink^™^ PCR Micro Kit (Invitrogen), quantified on a Qubit 2.0 fluorometer (ThermoFisher Scientific), and serially diluted in sterile TE to create a six-level standard curve dilution (6 250, 1 250, 250, 50, 10, and 2 copies per 4 μl). Each level of standard was run in six replicates for each assay.

We screened the assays *in vivo* against eDNA samples collected from eight sites along the Yellowstone River in Montana, USA (Table 4) for which the fish community assemblage was known from previous surveys. We collected these samples by filtering 5-l of water using a peristaltic pump following methods described in Carim et al. [26]. The samples were extracted with the DNeasy Tissue and Blood Kit (Qiagen, Inc) following a modified protocol [27] in a room dedicated solely to eDNA extraction. Extracted eDNA was stored at -20°C until qPCR analysis. Using the PCR recipe and optimized conditions above, we analyzed these eDNA samples in triplicate reactions with each assay and included a TaqMan Exogenous Internal Positive Control (Life Technologies) to monitor inhibition.

**Table 4:** Collection information for *in vivo* testing of the sauger and walleye eDNA assays. Samples were collected in the Yellowstone River, Montana. Detection expectation was determined from 2016 survey data.

				Expected/detected^1^	
Site	eDNA Collection date	Latitude	Longitude	Sauger^2^	Walleye^2^
1	9/23/2015	45.654233	-108.757244	N/N	N/N
2	9/23/2015	45.797100	-108.469000	N*/N	N/N
3	9/23/2015	45.999476	-108.128482	Y/Y	N*/N
4	9/23/2015	45.996292	-108.010161	Y/Y	N/N
5	9/23/2015	46.075410	-107.721700	Y/Y	N/N
6	10/28/2015	46.173200	-107.434600	Y/Y	Y/Y
7	10/28/2015	46.314700	-107.239600	Y/Y	Y/Y
8	10/28/2015	46.279800	-106.485200	Y/Y	Y/Y

^1^N (no) and Y (yes) refer to occupancy based on traditional surveys and eDNA-based detection.

^2^Asterisks indicate that fish were captured at a site ≥3 years prior but were not present in 2016 surveys.

## Results

The sauger assay successfully detected DNA in all 23 sauger tissue samples but not in any of the non-target samples or no-template controls. The standard curve experiment resulted in an efficiency of 95.78% (*r*^*2*^ = 0.99, y-intercept = 37.66, slope = -3.43) and the sauger assay had a limit of detection (defined as the lowest concentration with >95% amplification success; [28]) at 10 mtDNA copies per reaction. The sauger assay detected target DNA at concentrations of 2 copies per reaction in five of six replicates. Sauger DNA was detected in all environmental samples that were collected from areas known to contain sauger, and was not detected in any of the samples collected where sauger were expected to be absent (Table 4).

The walleye assay detected DNA from 18 of the 19 walleye tissues screened, and did not detect DNA in any of the non-target samples or no-template controls. The standard curve experiment resulted in an efficiency of 97.67% (*r*^*2*^ = 0.99, y-intercept = 39.25, slope = -3.38) and the walleye assay had a limit of detection at 10 mtDNA copies per reaction. The walleye assay detected target DNA at concentrations of 2 copies per reaction in five of six replicates. The walleye tissue that did not amplify originated in the Cumberland Drainage, Kentucky. To identify potential basepair differences between this individual and the walleye assay, we sequenced a 270-base region of the ND2 gene encompassing the assay location. Relative to sequences from our walleye specimens and those in GenBank, there were five base-pair mismatches within the forward primer, four within the reverse primer, and as many as 24 across the entire 270-base sequence. Because the sequence was nearly 10% different than any GenBank sequences, we sequenced a 652 base region of the cytochrome oxidase I and a 1,140 base region of the cyt*b* to confirm the sample was taken from a walleye. We performed a BLAST search with each sequence which resulted in a 100% match in both the COI and cyt*b* with a walleye collected in New River, VA (COI accession: KC819821.1, cyt*b* accession: KC819871.1; [2]). Walleye DNA was detected in all environmental samples that were collected from areas known to contain walleye, and was not detected in any of the samples collected where walleye are expected to be absent.

## Discussion

We have developed eDNA assays that reliably detect low concentrations of sauger and walleye DNA. Each assay is species-specific, detecting DNA from the intended targets and not from the non-target species we tested. The assays successfully detected DNA from environmental samples taken in central Montana where each species was expected to occur, and neither marker indicated the presence of a target species where it was expected to be absent. Using these markers, managers can quickly and reliably delimit the distributions of these species and prioritize conservation efforts throughout the northern U.S. In addition, sampling and analysis can be easily replicated over time providing an effective way to monitor temporal fluctuations in populations of sauger and walleye.

Given that our sauger eDNA marker accurately detected DNA of sauger from over 20 locations in the upper Missouri River drainage and matched all cyt*b* sequence data on GenBank (*n* = 19), including sequences of individuals from the mid-eastern United States and southern Canada, it would likely be adequate for detecting this species across its natural range. Nonetheless, with all eDNA applications, it is prudent to screen tissues of individuals from a given area of interest to verify that any genetic diversity present in the target species will not alter the sensitivity of the marker.

While our walleye marker reliably detected DNA of walleye from 14 locations across the United States and Canada, it did not detect DNA of walleye from the Cumberland drainage, the most southern population tested. White et al. [29] identified a distinct mitochondrial haplotype in walleye from the upper Cumberland River drainage that is divergent from northern populations (e.g. Great Lakes). This haplotype was also found in walleye from the upper New River in southwestern Virginia which Palmer et al. [30] suggest are ancestral to Ohio River populations. The unique lineage of walleye found in these areas is attributed to evolving in isolation in unglaciated rivers during the late Wisconsinan glaciations [31].

The ability of our walleye marker to distinguish between the northern and southern strains could be advantageous for management efforts aimed at protecting southern strains of walleye. Prior to the discovery of this genetically distinct lineage, populations of native walleye were thought to be extirpated from the upper Cumberland River drainage [32]. Throughout the 20^th^ century, the northern strain of walleye had been stocked in tributaries of the Cumberland River and in Lake Cumberland [32]. While hybridization between the northern and southern strains has been documented in some areas [33–34], the discovery of these genetically pure southern walleye prompted management activities to protect them as a unique lineage [32]. The stocking of the northern strain was halted and replaced with stocking of the native southern strain to encourage the re-establishment of genetically pure native walleye [32]. This marker can be used either to detect regions in which the northern strain is present, or to quickly determine whether a known population contains members of the non-native strain. Identifying areas where the northern strain of walleye remain present would help managers prioritize where conservation efforts for the native strain would be most effective.
